# Selections

**Published:** 1892-09

**Authors:** 


### *SELECTIONS.*

---

### THE MEDICAL TREATMENT OF CYSTITIS.

In the *Practitioner* Dr. Jas. Tyson contributes an interesting paper with this title:

The successful treatment of chronic cystitis is a much more difficult task. The constant presence of the urine in the bladder is very irritating to the inflamed mucous membrane. Diluents should be used freely, as in the treatment of acute cystitis. The mistake is sometimes made of adding alkalies to the fluid ingested, whereas the indication in chronic cystitis is to render the urine acid, if possible. Benzoic acid may be used for this purpose, but at least thirty grains a day must be given to produce any effect.

The vegetable acid should not be employed, as they are all eliminated as alkaline carbonates. After diluting the urine, the second indication is to medicate the inflamed surface. Two ways suggest themselves: (*a*) by the internal administration of drugs; (*b*) by the injection of medicated liquids into the bladder. Dr. Tyson has no confidence in the infusions and decoctions usually prescribed—such as buchu, pareira brava, uva ursi, and triticum repens. They simply act as diluents. The balsams, however, are really useful. Of these, the balsam of copaiba is practically unavailable, because very few stomachs will tolerate it in sufficient doses.

Sandal-wood oil is of great service, and in Dr. Tyson's experience is the only remedy that has any direct effect on the mucous membrane of the bladder. It is usually well borne by the stomach, and is best administered in capsules containing ten minims. Eight capsules should be given each day—two before each meal and two at bedtime.

Boric acid and benzoic acid are both useful adjuvants to the treatment of chronic cystitis by reason of their antiseptic effect on the urine, each in five-grain doses, rapidly increased to ten.

Resorcin may be used in the same doses, and naphthaline in doses of two grains.

The application of remedies to the bladder by injections may be considered in connection with a third indication—namely, the getting rid of the products of inflammation, the pus and mucus, and the compounds resulting from their decomposition.

This can only be done by washing out the bladder. Tepid water should be used first, and the injection made through a soft catheter. Four ounces may be thrown in at a time and then allowed to run out. This operation should be repeated until the water comes out clear.

After a few injections with plain water some medicament may be added. Dr. Tyson prefers salicylate of sodium in the proportion of a drachm to the pint. Boric acid, in the same proportion, is also very satisfactory. Alum may be used instead of salicylate of sodium when the pus does not diminish so rapidly as is desired. When there is a foul odor present, bichloride of mercury, in a very dilute solution, is of service. One should not begin with a solution stronger than 1 to 25,000; if this is well borne, the strength may be increased gradually.

Anodynes are indispensable in many cases to relieve the patient of extreme pain and the frequent desire to pass water. Opium and its alkaloids are the most efficient, and are best introduced by the rectum.—*N. Y. Med. Journal.*

---

### DRUGS, ESPECIALLY THE HYPOPHOSPHITES, IN THE TREATMENT OF EARLY PHTHISIS.

In a paper read at the recent meeting of the British Medical Association, Thorowgood (*British Medical Journal*, October 17, 1891) discusses the use of drugs in the treatment of early phthisis. The discovery of the tubercle bacillus has enabled us to differentiate the acute tubercular form of the disease from that catarrh of the apex due to cold or to long continued respiration of close, vitiated air, and associated essentially with a degenerated condition of the epithelium. In these latter cases change to a purer atmosphere is productive of the greatest benefit. When, however, the patient's circumstances do not permit of change of surroundings, reliance must be placed upon drugs alone, and here the author has had gratifying results with the hypophosphites. Five grains of hypophosphite of soda in water or infusion of calumbo three times daily for five or six weeks have completely cleared up catarrhal conditions of the apex left after bronchitis, and consolidations after pneumonia, as well as pleuritic exudations remaining after a pleurisy. In cases of phthisis attended with fever and rapid emaciation, which in former days the author recognized as beyond the help of drugs, he now believes the tubercle bacillus was too strong to be overcome by a medicine whose action lay mainly in promoting absorption of inflammatory products. The hypophosphites, or phosphorus itself, which he thinks inferior to its salts, act by a process of fatty change and liquefaction. When this process seems to be attended with some increase of temperature, the dose should be reduced or should be given at longer intervals. In recurring hæmoptysis, also, the hypophosphites must be used with care. The potash salt is the most active in liquefacient power, while the lime hypophosphite often acts remarkably well where secretion is profuse, and checks excessive sweating and diarrhœa. The dose should not exceed five grains, and is best given with five or ten drops of the saccharated solution of lime, glycerine, and sometimes syrup of tolu. Passing over other drugs, the author speaks well of the inhaling respirator, especially with iodoform and eucalyptus oil. Persistent and even severe counter-irritation by croton oil or liniment should not be forgotten as a valuable adjunct in treatment.—*Univ. Med. Mag.*

---

### THE DECADENCE OF ANTISEPTICS.

We believe, from what can be gathered from the latest literature on the subject, that antiseptic surgery, as taught and practiced as recently as five years ago, is now something of the past.

When Lister introduced the spray, and argued with great force the importance of purifying the atmosphere of the operating room, and seeking out the stray germs lodged in sundry recesses of one's apparel or surroundings, Lawson Tait, with stinging sarcasm, recommended that the nozzle of the apparatus would do the most good when pointed out through the window. The spray went.

Reports commenced to come in that antiseptics must be eschewed in the surgery of the peritoneum. It was discovered that traumatisms penetrating the skull and involving the brain, when treated antiseptically, were attended with a terrible mortality through a consecutive, irritative meningitis. Antiseptic irrigation of the pleura in empyema, is no longer employed by the French surgeons.

Bichloride solutions, when used in amputations, though they favor prompt union, are said to cause very often painful, useless stumps, through an insidious osteo-myelitis which they excite in the cellular elements of the cancellous bone substance.

Antiseptics—or, rather, chemical solutions—were practically condemned by the American Surgical Convention of 1891. Prof. Chiene, a townsman of Lister, sounded the death-knell of antiseptics in Great Britain when he announced and demonstrated that chemical solutions of any description were foreign substances, irritants, and had no place in healthy tissues.

This, indeed, is a sad commentary on what was taught but yesterday as a cardinal doctrine.

Morrell Mackenzie narrowly escaped imprisonment at the hands of his unfriendly German confrères because he did not boil or pickle his spatula every time he used it on the Prince's tongue.

Although the above must be a humiliation to those who pin their faith absolutely to scientific medicine, it will teach a useful lesson to many who are too strongly inclined to dogmatize.—*Times and Register.*

---

### CAMPHORIC ACID FOR NIGHT-SWEATS.

Dr. H. A. Hare, in the *Therapeutic Gazette*, highly recommends this drug for the condition named. The acid is a white, micaceous, powdery substance, devoid of odor, and almost of taste. It is, unfortunately, practically insoluble in water, and must be given in pills, capsules or compressed tablets. Originally introduced into medicine as an insufflating powder in cases of coryza and hay-fever, I have found it of the greatest possible value in night-sweats, be their cause what it may. It is in night-sweats of phthisis that I have found it most useful. Any one who has had many of the cases to treat will have had repeated failures from the older antihydrotics. In my earlier terms of service at St. Agnes's Hospital I employed sulphuric acid, belladonna, atropine, minute doses of pilocarpine, and a number of similar remedies, with one of several results. Either relief was obtained temporarily, the patient having a comfortable night or two before the medicine failed him, or, more frequently, the dominant action of the drugs upon other portions of the body than the sweat-glands was so strongly manifested that the dryness of the throat, the belladonna wakefulness, or the pilocarpine nausea prevented the administration of large enough doses to be effective.

Camphoric acid, under these circumstances, proved to be a most efficient drug, for it has stopped the sweats in every case ; in but one out of sixty cases in which I have used it has it failed, and it has become as constant a mode of treatment as is the cough-mixture to allay excessive cough. I would as readily undertake the treatment of a case requiring an anæsthetic without ether or chloroform as one of phthisis without camphoric acid. I will not weary you with reports of cases ; suffice it to state that the drug should be given in one full dose two hours previous to the expected sweat, being placed dry on the tongue, and washed down with water or wine, or it may be given in capsule. If any diarrhœa is present, compressed tablets cannot be used, as they will be swept out of the alimentary canal before their contents can be absorbed. The average dose is twenty grains, but I have frequently given sixty grains at one time without bad effect, and on one occasion as much as one hundred and eighty grains a day without injury to a frail woman. Further than this, I have never seen it cause a symptom which would show that it had been given, save the arrest of the sweating.

### SOME GOOD FORMULAS FOR DANDRUFF, ETC.

Dr. J. V. Shoemaker, Philadelphia, recommends the following:

℞. Balsam. Peru........................ʒ ss.

Beta-naphthol........................ʒ j.

Lanolin............................ʒ vj.

Adipis benzoinat.....................ʒ ij,

M. ft. ungt.
For dandruff.

℞. Tr. cinchon. co.....................f ℥ j.

Tr. benzoin. co......................f ℥ ij.

Glycerin.............................f ʒ j.

Sp. odorat,

Aquæ.............................āā f ℥ ij.

M. For dandruff.

℞. Potass. carb.........................ʒ j.

Aq. ammon...........................f ʒ vj.

Tr. cantharid........................f ʒ iiss.

Ol. myrist...........................gtt. xii.

Sp. odorat..................q. s. ad O ss.

M. ft. sol.
For dandruff.

℞. Hydrarg. chlor. corros............ gr. xv.

Glycerini..........................f ʒ ij.

Sp. myrciæ.........................f ℥ iv.

Ol. geranii..........................*m* xvj.

Aquæ.......................q. s. ad O ss.

M. ft. sol.
For general thinning and loss of hair.

℞. Salol............................ʒ ss

Acid. tannic.........................ʒ j.

Balsam. Peru........................℥ ss.

Lanolin............................℥ ss.

Adip. benzoinat.......................ʒi

M. ft. ungt.
For dandruff.

℞. Acid. borici..................... ........℥ ss.

Hydrarg. chlor. corros..................gr. xx.

Glycerini,

Aquæ...............................āā f ℥ iv.

M. ft. sol.

For loss of hair.

. Acid. borici............................ʒ ij.

Glycerini................................℥ ij.

Sp. vini gall............. .............℥ iv.

M. For general thinning and loss of hair.

—*The Medical Bulletin.*

---

### UNCURED GONORRHŒA IN THE FEMALE.

A few words about uncured gonorrhœa in the female. Who can surely say when a woman is cured of a clap, or what is perhaps of more importance, who can declare by any means at his command that a suspected woman is free of the disease? How commonly is gonorrhœa contracted by some one from a subject who declares herself innocent of the disease, and points to a healthy vagina and frequent harmless congress with other men in proof of her claim. How often does the one child mother, with her history of pelvic phlegmon and her painful, fixed, and deflected womb, demonstrate the invasion of the deeper organs by an old specific vaginitis through os and cervix made patulous by her parturition. Again and again have I expressed with a bivalve speculum through an apparently normal os into a pale, cool, dry vagina, a quantity of muco-purulent matter of specific character. Who then can tell of the possibilities of infection that lurk still further up in womb and appendage? It is not even necessary, as in the case of the male, to conjure up a relighting of anterior inflammation, a self-infection brought about by wine and the dance, for may not each menstruation serve as an agency to bring down the lurking virus for man's infection? Here, more than in any other way, to my mind, is found explanation of the commonly recognized danger of infection by sexual congress during the molimen. Surely something more than menstrual blood and a crumbling but normal endometrium must be looked to for the cause of this not infrequent catastrophe. In conclusion let me say, as of the women even more than of the men, gonorrhœa is frought with decidedly more danger than is syphilis.—*Dr. E. R. Palmer in Am. Pract. & News.*

---

### TREATMENT OF BUBOES.

McBurney divides his cases under three classes; those in which the glands are tender and hard without any sign of septic infection; those in which there are signs of gland deterioration, tenderness, redness, and softening; those in which the glands are completely broken down. Cases of the first class are treated by the application of cold. Those of the second are suitable for excision, all the glands being removed, and those portions of the skin which are badly involved in the inflammatory process. This treatment succeds in proportion to the freedom of the glands from softening. It is in the third class of cases, in many of which excision is hopeless, that he has been led to adopt injections of iodoform and vaseline, as advised by Pontain. He has treated twenty cases. The method pursued was to make a small opening, through which the fluid is expressed. A small spoon is then introduced and dividing septa and connecting tissue bands broken down. The sac is then overdistended six to twelve times with a bichloride solution 1 to 1,000. Then by means of a syringe a mixture of iodoform and vaseline, a drachm to the ounce, which has been heated to fluidity, is injected. The application of a cold gauze compress causes the vaseline to harden in a few minutes. In two cases treated by him complete failure has resulted; in eight a partial success was secured, and in the remaining ten the success was complete after the first or second injection. Five had recovered before the fourteenth day, and the average duration of all was nineteen days.—*Times & Register.*

### THE SALOL TREATMENT OF ACUTE AND CHRONIC CYSTITIS.

Dr. B. Arnold (Stuttgart) has employed salol in cystitis and deduces as follows:

1. Salol makes the alkaline urine acid.

2. It removes the foul odor.

3. It clears the cloudy urine, the muco-purulent sediment is diminished, and becomes lighter and flocculent, disappearing finally entirely.

4. The amount of urine is generally increased.

5. The stomach retains this remedy very well, and for a considerable time; this makes salol superior to other drugs employed in cystitis.

This property is probably due to the insolubility of this drug in the gastric juice, becoming solved only after it reaches the intestines and the pancreatic juice is added, dividing it in salicylic acid and phenol (*Rosenberg*).

6. It is an excellent adjunct in washes for the bladder, especially when only weak antiseptic solutions are borne.

Arnold does not claim salol as a panacea, but as an excellent remedy in acute and chronic cystitis, rivaling the best anti-cystitis remedies.—*Therap. Monatshefte.*

---

### INTERRUPTION OF CIRCULATION IN EXTREMITIES FOR ANÆSTHESIA.

Dr. McBurney advises shutting off the circulation in the extremeties (upper and lower) before a patient is to be anæsthetized. He claims that the effect of the anæsthetic is much more prompt, that vomiting is avoided almost with a certainty, that complete recovery from the drug takes only a few minutes after the loosening of the tourniquets. If hypodermic injections (morphia) are given their dose must be much smaller than under ordinary circumstances. An additional advantage is the perfect rest of the limbs which under the usual plan frequently annoy the surgeon by movements and trembling.—*Medical Age.*

### THE TREATMENT OF PLEURITIS.

Fiedler refers to the etiological identity of rheumatic polyarthritis and certain forms of acute pleuritis, as a guide for the favorable action of salicylic acid in such cases. He has used salicylic acid for years in cases of pleuritis where no tuberculosis was shown to exist, and is convinced from long experience, that if it is given early enough before the appearance of the exudation, the disease may be aborted. Large doses, up to 90 grains of the salicylate of soda, are administered. Little is to be expected from the salicylate after the occurrence of an exudation. He is also convinced of the utility of this preparation in genuine cases of pericarditis.—*Münchner med. Wochenschrift.*

Dr. Talamon directs attention to the efficient action of salicylate of sodiumin the treatment of pleurisy with effusion. He reports cases showing how, after the administration of the drug, rapid resorption of the effusion occurred. In three of the cases thoracentesis had already been twice performed, but the fluid had accumulated as abundantly as before. The salicylate of sodium was then given with the result that, at the end of a week, the effusion had disappeared. A diminution of fluid was noted on the second day of the treatment. He does not attribute the action of the salicylate to its diuretic properties, for while it may increase the quantity of urine, diuresis can also be provoked by other drugs, and yet the pleural effusion remains the same. The salicylate of sodium, according to the author, should be given for a week, the dose being 15 grains, four to six times a day. The more recent the pleurisy the better the action of the drug. It is especially indicated, after thoracentesis, to complete the resorption of the exudation and prevent its reaccumulation.—*Médecine Moderne.—Occidental Med. Times.*

---

### THE PROPHYLAXIS OF SYPHILIS.

In his new work on the Practice of Medicine, Dr. Osler thus says:

Personal purity is the prophylaxis which we, as physicians, are bound to advocate. Continence may be a hard condition (to some harder than to others), but it can be borne, and it is our duty to urge this lesson upon young and old who seek our advice in matters sexual. Certainly it is better, as Paul says, to marry than to burn, but if the former is not feasible there are other altars than those of Venus upon which a young man may light fires. He may practise at least two of the five means by which, as the physician Rondibilis counselled Panurge, carnal concupiscence may be cooled and quelled—hard work of body or hard work of mind. Idleness is the mother of lechery; and a young man will find that absorption in any pursuit will do much to cool passions which, though natural and proper, cannot in the exigencies of our civilization always obtain natural and proper gratification.

---

### SPUTUM AS A DIAGNOSTIC SIGN.

In phthisis we have nummular sputum; looks like coin, which floats in a clear liquid.

In measles we have nummular sputum, which floats in an opaque liquid.

In bronchiectasis there is stinking sputum; also in fibroid phthisis we have stinking sputum.

In cancer of the lung we have sputum that looks like currant jelly.

In pneumonia we have rusty colored sputum.

In edema of the lung the expectoration is serous.

Where we have pneumonia terminating in gangrene of the lungs, the sputum is exceedingly fetid; greenish or brownish.

The sputum of chronic bronchitis, when associated with disease of the heart, looks like the white of egg mixed with water, and may amount to a quarter or half gallon in twenty-four hours.

The sputum of chronic bronchitis, when not complicated, is large, broad and irregular, and is greenish or yellowish.—DR. MORRIS, *Times and Register*.

### SYPHILIS.

Of the use of mercury Professor Fournier writes: As to the dose on which success depends, you must not be afraid. What is the most useful quantity? I regret that I have to say that most physicians keep below the proper quantity, as I learn in consultations I have with them. Let me give you an example; I was asked to see a fine, big fellow whom I found with a tubercular syphilide, and who three years before had been treated for syphilis for a few months, during which he took only $\frac{1}{6}$ grain of proto-iodide of mercury per day, which, for such a big man, was nothing at all, so that during three years he had his syphilide. I have notes of another case that was treated with $\frac{1}{60}$ grain of corrosive sublimate per day, and now has phagadæna. So you see that many patients are not treated properly." This is a sort of "disguised expectation." It is a delicate matter, I know, absolutely to fix a dose of mercury in syphilis. There is no exact dose; the curative dose of corrosive sublimate for a man is about ½ grain; for a woman it is ⅓ grain; below this the drug has no active effect on the disease. The dose of the proto-iodide is larger; for a man about two grains per day, and for a woman 1⅓ grains. This is the most efficacious dose, capable not only of meeting all the important indications, but also of preventing symptoms that might arise in the future.—*Univ. Med. Mag.*

---

### FOR SCIATICA.

Dr. Starr, in his work on "Nervous Diseases," gives the following as having proved useful in his practice in the treatment of sciatica. Anything that holds out a hope of relief in this *bête noir* of the profession may be welcomed.

℞. Tinct. colchici.......................................*m* ¾

Tinct. cimicifugee....................................*m* ¾

Tinct. aconiti..........................................*m* ¾

Tinct. belladonnæ....................................*m* ¾

Sig.—One dose.—*Memphis Med. Monthly.*

### PROPHYLAXIS OF DIPHTHERIA.

In the *Medical and Surgical Reporter*, Dr. G. Betton Massey says: During the ten years that I spent in general practice I invariably prescribed insufflation of powdered sulphite of sodium in those cases of diphtheritic sore throat, with general systemic disturbance, that seemed to be the first stage of diphtheria. In all these cases the membrane disappeared in from twelve to twenty-four-hours, and it was a curious fact that in these ten years I did not see a bad case of diphtheria. Whether these membranous sore-throats were of that nature, and were arrested, I am, of course, uncertain. The powder was insufflated through a paper roll every half hour, the paper being burnt after each insufflation, to prevent the wrong end being subsequently used and infecting the nurse.

---

### DISORDERED CONDITION OF THE PRIMÆ VIÆ.

Dr. J. M. Hays, of Oxford, N. C., uses the following for a furred tongue—large and flabby, with a bluish cast—anorexia, constipation, perhaps alternating with diarrhœa, slight headache, general lassitude, sleeplessness, loss of interest in business and pleasure, etc.:

℞. Podophyllin.................................gr. x.

M. Tinct. nux. vom............................ ℥j.

Sig.—Shake and take from five to eight drops in a little water before each meal.—*Brooklyn Medical Journal.*

---

### FISSURED NIPPLES.

℞. Balsam of Peru,
Tr. arnica........................................aa ℥ss
Oil of sweet almonds..........................℥j.
M. Lime water.......................................℥ss.

Sig.—Apply a small quantity several times daily.—VERRIER.
—*Memphis Med. Monthly.*

### ARISTOL IN CUTANEOUS DISEASES.

The results obtained from the use of aristol by Dr. Iginio Sormani, in the Opededale Maggioro, in Milan, are very satisfactory, and even far surpass expectation. Some of the cases had been previously under treatment without deriving any material benefit. Of especial importance is a case of *ulcerating epithelioma* extending from the ala nasi to the eye (the diagnosis was confirmed by microscopical examination). Concentrated solution of resorcin had been employed without noticeable improvement; and the thermo-cautery and curetting were also unsuccessful. As early as six days after the application of a ten per cent. ointment of aristol cicatrization set in, and after thirty-five days a firm cicatricial tissue had formed. *Ulcerating lupus* and *scrofuloderma* were treated with aristol with much success, although a number of well-known remedies had been previously tried without avail. A case of extensive *ecthyma of the leg* in a man is also reported, in which the eruption could be made to disappear by the use of calomel, but constantly recurred; after the application of an aristol ointment, however, a formation of firm permanent cicatricial tissue took place. Numerous cases of *ulcer of the leg* were also treated successfully with aristol. Sormani concludes that on the ground of his experience and that of others, he regards aristol as an excellent remedy, which, in many respects, is superior to iodoform. It can be readily applied, and *is non-poisonous*, as is shown by the fact that even after prolonged application of aristol to extensive ulcerations no iodine can be demonstrated in the urine. We are, however, not as yet in a position to dispense with the other remedies and make use exclusively of aristol.—*Bolletino Della Poliam Bulanza.*

---

### THE TREATMENT OF ASTHMA.

The treatment of *Asthma* Prof. Da Costa divides into three heads. First, that *for the paroxysm:* Give a plentiful supply of fresh, pure air and some remedy to relieve the spasm—ether (internally, twenty drops) or chloroform or chloral; also dry cupping the chest and some slightly nauseating agent which will promote expectoration. Apomorphia is very useful; so are the salts of ammonium. Other remedies that may be used for the spasm are to burn paper that has been saturated with nitrate of potassium (nitre paper), inhalation of steam, and strong, hot coffee.

Secondly, remedies *to prevent the paroxysm:* Smoke stramonium. This is undoubtedly effective, and may be smoked in an ordinary pipe or thrown on a hot coal and inhaled. Belladonna is useful, as is also the combination of belladonna, camphor, and stramonium in the form of cigarettes. Nitrite of amyl may prevent it, but cannot be depended on.

Thirdly, the treatment *to get rid of the disease*: Change of air; each case is a law to itself. Colorado and New Mexico are the best climates. The food should be plain and no heavy suppers. The bowels should be kept open and the patient should be dressed warmly. Remedies that may be used are arsenic, which is excellent; nitroglycerine, ($\frac{1}{200}$–$\frac{1}{100}$ gr. three times a day) and the nitrites; preparations of iodine (iodide of sodium) in cases of lung complications. Blisters are of no use. It is very important that the patient's urinary secretions should be looked after.—*College and Clinical Record.*

---

### INTRA-UTERINE INJECTIONS.

Tarnier (*Jour. des Sages Femmes*) has determined never to employ sublimate lotions for intra-uterine injections. Eighteen cases of death, after sublimate injections, in childbed, have been recorded; in sixteen of these cases the injections were thrown into the uterus, in two only into the vagina. Death may be due to some severe reflex stimulus, or to direct poisoning through entrance of the injected fluid into the uterine veins. From experiments, it seems that permanganate of potassium, microcidine, iodine, and salicylic acid are innocuous. Sublimate is liable to involve dangers some time after its injection. Of substances which may cause syncope or immediate death when injected, carbolic acid holds first place. Biniodide of mercury is also very dangerous, and the perils of perchloride of iron are well known. Tarnier reminds the obstetrician that perfectly innocuous solutions, or even plain water, have caused death when injected into the veins. Any kind of injecting apparatus may prove dangerous if the obstetrician or nurse neglect to drive the air out of the tube, or uses too great propelling force. When gravitation is the agent, the receptacle for the fluid should not be placed more than fifteen inches above the level of the patient's pelvis.—*Archives of Gynecology.*

---

### Convulsions in Children.

Prof. J. E. Winters (*Med. Rec.*) says: Safe rule in all cases to see that stomach and alimentary canals are emptied. For this, give a full dose of ipecac, repeating in ten minutes if necessary. If this fails to bring on vomiting, tickle the fauces till successful. Give a copious enema, and follow with a half-ounce dose of castor oil. After purging, give chloral till relief is obtained. For a child two years of age, ten grains in a little water can be given per rectum. A child two years of age will stand half the quantity. Morphine may be used if the convulsions are due to uræmia. If scarlet fever or measles are coming on do not purge too freely, as it delays the eruption. The popular hot bath should be used with caution. If the cause of the convulsions is the advent of an infectious disease it may do good, as it brings the blood to the surface of the body and relieves internal congestion. If the convulsions are due to central lesions when treatment is of no avail, the hot bath may possibly be used for the consolation of the parents.—*Archives of Gynecology.*

---

### Agaricin in Night Sweats.

Few practitioners, says *Technics*, appreciate the exceedingly great value of agaricin as a remedy in night sweats, especially those of phthisis. The most profuse sweat is checked almost like magic, with a single dose. It operates by diminishing thirst and increasing the secretion of urine. The dose may be pushed to the extent of one grain in the course of twenty-four hours. The single dose for an adult is from one-eighth to one-fourth of a grain.—*West. Med. Reporter.*

### MALARIAL HÆMATURIA.

This condition, to which M. would give the name *Lysœmia*, occurs only in persons suffering from chronic malarial toxæmia. Their blood has deteriorated, and has a tendency to disintegration of the red corpuscles. The walls of the capillaries are weakened by malnutrition, and local disease of blood pressure, such as occurs in the internal organs during a chill, is followed in the kidney by choking up of the tubules with disintegrated corpuscles. Hæmaturia and suppression of urine follows, and unless speedily relieved, coma and death supervene. Quinine is useless or injurious. The indications for treatment are: 1, to clear up the urine; 2, to evacuate the bowels; 3, to repair the blood and blood-vessels; 4, to administer an antimalarial which will not interfere with the other indications. The first indication is met with turpentine, ten drops every four hours; Epsom salts supplies the second; the third is met with nourishment, mainly milk and iron; for the fourth arsenic is preferred.—DR. E. H. MARTIN, *in New Orleans Med. and Surg. Jour.*

---

### PLEASANT COUGH-MIXTURE.

℞. Ac. hydrocyan. dil............................*m*iij.

Syr. picis liq.,

Syr. prun. virg..............................āā ℥ss.

Spts. menth. pip..................................*m*x.

Syr. simp..............................q. s. ad ℥ij.

M. et Sig.: Half to teaspoonful every two or three hours.

The mixture is quite pleasant to take and will promptly check a cough without producing nausea.—BALLARD, *in Miss. Med. Monthly; St. Louis Clinique.*

---

### THE LAW AS REGARDS TWINS.

When twins are born in France, the last born is considered by law the eldest! Consequently, if both survive, and, in the case of boys, reach manhood, the second is called to the army to serve, being pronounced the eldest. By some extraordinary calculation the medical men who were consulted at the passing of the act years ago came to the conclusion that the last born of twins was always the first conceived.—*Medical Press and Circular.*

